# Orbitofrontal cortex, emotional decision-making and response to cognitive behavioural therapy for psychosis

**DOI:** 10.1016/j.pscychresns.2015.01.013

**Published:** 2015-03-30

**Authors:** Preethi Premkumar, Dominic Fannon, Adegboyega Sapara, Emmanuelle R. Peters, Anantha P. Anilkumar, Andrew Simmons, Elizabeth Kuipers, Veena Kumari

**Affiliations:** a Division of Psychology, School of Social Sciences, Nottingham Trent University, Nottingham, UK; b Department of Psychology, Institute of Psychiatry, Psychology and Neuroscience, King׳s College London, London, UK; c NIHR Biomedical Research Centre for Mental Health, South London and Maudsley NHS Foundation Trust, London, UK; d South London and Maudsley NHS Foundation Trust, London, UK; e Department of Neuroimaging, Institute of Psychiatry, Psychology and Neuroscience, King׳s College London, UK

**Keywords:** Asymmetry, Prefrontal cortex, Schizophrenia

## Abstract

Grey matter volume (GMV) in the orbitofrontal cortex (OFC) may relate to better response to cognitive behavioural therapy for psychosis (CBTp) because of the region׳s role in emotional decision-making and cognitive flexibility. This study aimed to determine the relation between pre-therapy OFC GMV or asymmetry, emotional decision-making and CBTp responsiveness. Emotional decision-making was measured by the Iowa Gambling task (IGT). Thirty patients received CBTp+standard care (CBTp+SC; 25 completers) for 6–8 months. All patients (before receiving CBTp) and 25 healthy participants underwent structural magnetic resonance imaging. Patients׳ symptoms were assessed before and after therapy. Pre-therapy OFC GMV was measured using a region-of-interest approach, and IGT performance was measured as overall learning, attention to reward, memory for past outcomes and choice consistency. Both these measures, were comparable between patient and healthy groups. In the CBTp+SC group, greater OFC GMV correlated with positive symptom improvement, specifically hallucinations and persecution. Greater rightward OFC asymmetry correlated with improvement in several negative and general psychopathology symptoms. Greater left OFC GMV was associated with lower IGT attention to reward. The findings suggest that greater OFC volume and rightward asymmetry, which maintain the OFC׳s function in emotional decision-making and cognitive flexibility, are beneficial for CBTp responsiveness.

## Highlights

• More orbitofrontal cortex volume relates to cognitive behaviour therapy for psychosis responsiveness.
• More rightward OFC asymmetry relates to cognitive behaviour therapy for psychosis responsiveness.
• More orbitofrontal cortex volume relates to better emotional decision-making in schizophrenia patients.

## 1 Introduction

Multiple lines of enquiry suggest that the effectiveness of cognitive behaviour therapy (CBT) in many disorders, including schizophrenia, is associated with the integrity of neural structures that support specific cognitive processes (Brody et al., 1998, Premkumar et al., 2009, Yamanishi et al., 2009). The function of the orbitofrontal cortex (OFC) may be particularly important in this context, since it has been shown that better pre-therapy OFC function, in terms of metabolic activity and cerebral blood flow, relates to better CBT responsiveness in patients with obsessive–compulsive disorder (Brody et al., 1998, Yamanishi et al., 2009).

Greater OFC GMV may relate to responsiveness to CBT for psychosis (CBTp) because of its role in emotional decision-making and cognitive flexibility (Bechara et al., 1994, Bechara et al., 2000, Schoenbaum et al., 2009). Emotional decision-making is the ability to respond with emotional sensitivity contingent on the reinforcement consequences of the stimulus (Rolls and Grabenhorst, 2008). Making decisions about engaging in risky or safe behaviour requires emotional regulation (Bechara et al., 1994). Good emotional decision-making implies being able to successfully regulate one׳s emotions while under pressure to make risky decisions, such as deciding whether a deck of cards will lead to monetary gain or loss. It can reflect a decision-making style that is more rational and controlled, and less impulsive (Patrick et al., 2008). The Iowa Gambling task (IGT) is a frequently used measure of emotional decision-making (Bechara et al., 1994, Bechara et al., 2000). The IGT is a task that requires participants to choose a card from four decks that differ in the amount of monetary gain or loss they offer to participants as a result of making that card choice. Individuals with OFC damage show impaired emotional decision-making (Hornak et al., 2003, Bechara, 2004); their performance is motivated by reward value, and they are oblivious to losses. Schizophrenia patients, on average, show an IGT performance style that is similar to that seen in patients with OFC lesions (Shurman et al., 2005, Nakamura et al., 2008).

Another possible reason for the relationship between OFC volume and CPTp responsiveness is that reduced OFC volume relates to higher impulsivity in schizophrenia patients, a personality characteristic suggestive of lower cognitive flexibility (Kumari et al., 2009a, Schiffer et al., 2010). Schizophrenia patients are known to have a jumping-to-conclusions reasoning style (Garety and Freeman, 1999, Garety et al., 2005) and poorer cognitive flexibility (Ross et al., 2011). CBTp for persistent positive symptoms targets reasoning biases (Kuipers et al., 2006, Waller et al., 2011, Freeman et al., 2012). The OFC is involved in the ability to respond flexibly to situations where one must change an established behavioural response in order to adapt to new contingencies (Schoenbaum et al., 2009) and update information about the reward value of specific outcomes to influence subsequent behaviour (Gottfried et al., 2003). Moreover, greater OFC volume relates to having better clinical insight, in terms of having less likelihood of misattributing symptoms in patients with first episode psychosis and chronic schizophrenia (Shad et al., 2006, Sapara et al., 2007). Having better foresight – the ability to think of the long-term consequences of one׳s behaviour and use this information to guide present and future actions – is associated with greater OFC volume in schizophrenia patients (Eack et al., 2008).

The volumetric asymmetry of the OFC between the two hemispheres may also be relevant to treatment response in schizophrenia patients (Robinson et al., 2004, Szeszko et al., 2012). Greater rightward OFC asymmetry was associated with lower overall symptom severity in first episode patients (Sheng et al., 2013). Nonresponders to antipsychotic drug treatment lacked middle frontal asymmetry compared with responders (Szeszko et al., 2012). Thus, existing data about the clinical correlates of OFC asymmetry indicate that greater rightward OFC asymmetry is conducive for better clinical outcome, possibly because of better OFC interhemispheric functional connectivity.

The aim of the current study was to determine whether (a) patients receiving CBTp would differ from healthy individuals in pre-therapy OFC GMV, (b) greater pre-therapy OFC GMV/asymmetry would relate to CBTp responsiveness in patients with schizophrenia or schizoaffective disorder, and (c) greater pre-therapy OFC GMV/asymmetry in patients would relate to better IGT performance. We anticipated that patients would have normal or near-normal emotional decision-making, because IGT performance, on average, was normal in a larger sample of patients from which the present patient cohort was drawn (Premkumar et al., 2008). Based on the literature reviewed above on the OFC׳s role in cognitive flexibility, we hypothesized that (1) greater pre-therapy OFC GMV would relate to positive symptom improvement following CBTp, (2) greater pre-therapy OFC GMV would relate to better IGT performance, and (3) greater rightward OFC asymmetry would relate to better CBTp responsiveness.

## 2 Experimental procedures

### 2.1 Participants and design

This investigation was carried out as part of a larger project (Kumari et al., 2009b, Kumari et al., 2011, Kumari et al., 2012) which involved 60 outpatients with a DSM-IV schizophrenia or schizoaffective disorder diagnosis. The outpatients were recruited from the South London and Maudsley NHS Foundation Trust and were willing to receive CBTp. Half of these 60 patients received CBTp, in addition to standard care (SC), during the course of this project. The patient sample for this study consists of 25 of these 30 patients (one patient discontinued therapy and four patients withdrew consent) for whom we fully completed clinical assessments at baseline and follow-up. Patients were on a stable dose of antipsychotic drugs for at least 2 years and their current antipsychotic drug for at least 3 months. Medication was mostly in the form of second generation antipsychotics. The patients had an illness duration (defined as duration since first hospitalization) of on average 11.13±7.88 years and were on average 24.44±7.99 years old at the time of first hospitalization. Twenty-two patients had paranoid schizophrenia, two patients had schizophrenia with subtype not specified, and one patient had schizoaffective disorder. The level of antipsychotic medication at baseline was 520.72±380.70 (in mg chlorpromazine equivalents). To be included in the study, patients had to have scored 60 or more on the Positive and Negative Syndrome Scale (PANSS) (Kay et al., 1987) and have one or more distressing positive symptoms, i.e., score 3 or more on a PANSS positive subscale item. In addition, CBTp+SC patients were those who were referred to and accepted for CBTp by the Psychological Interventions Clinic for Outpatients with Psychosis (PICuP) within the South London and Maudsley NHS Foundation Trust. Twenty-five healthy participants were also drawn from the local community through advertisements. Participants were excluded if they had a previous diagnosis of neurological or organic illness, head injury or a concomitant diagnosis of drug or alcohol dependence. All patients (before receiving CBTp) and healthy participants underwent structural magnetic resonance imaging (MRI) and were assessed on the IGT.

The study was approved by the ethics committee of the joint research ethics committee of the Institute of Psychiatry, Psychology and Neuroscience and the South London and Maudsley NHS Foundation Trust, London. All participants provided written informed consent.

### 2.2 CBTp and standard care

CBTp was based on the model of intervention described by Fowler et al. (1995), where the aim was to reduce distress and interference arising from psychotic symptoms; reduce depression, anxiety and hopelessness; and modify dysfunctional schemas when appropriate. It was based on individualized formulations. CBTp was delivered weekly or fortnightly (as preferred by the patient) over an average of 16 individual 1-h sessions. Therapy lasted for 6–9 months. Initial sessions focused on facilitating engagement in therapy. The therapist tried to build and maintain a good therapeutic relationship by taking a flexible approach that focused on the patient׳s needs (Kuipers et al., 1997). The therapists were all qualified CBT practitioners and were supervised by experienced clinical psychologists with expertise in CBTp (E.R.P. and E.K.). The PICuP specializes in this intervention. Therefore, no other intervention was provided at the PICuP.

Standard care (SC) consisted of case management offered by the case management team for a particular geographical area. The team included a dedicated care coordinator who saw the patient on a regular basis. The frequency of meetings with the patient ranged from weekly to monthly, depending on how stable the patient was. In addition, the patient had regular reviews with a psychiatrist and other specialists such as a psychologist, vocational adviser and occupational therapist as needed. Six-monthly care plan assessment reviews were carried out with a focus on recovery.

### 2.3 Clinical assessments

Clinical diagnosis was confirmed by the Structured Clinical Interview for DSM-IV (First et al., 2002). PANSS assessments (Kay et al., 1987) were performed on all patients before and after CBTp by an experienced consultant psychiatrist (D.F.). This psychiatrist played no role in patient recruitment or clinical management of any patient included in this investigation. The PANSS three-factor model (Kay et al., 1987) was used to measure the severity of positive and negative symptoms and general psychopathology.

### 2.4 Handedness

The Edinburgh Handedness Inventory measured handedness as the total out of a maximum of 10 actions for which the participant had a right-sided preference when performing routine action (Oldfield, 1971). The Grooved Pegboard test (Matthews and Klove, 1964) is a motor dexterity test. Participants were instructed to place 25 pegs as quickly as possible into separate slots, each with a different orientation, while moving consecutively row-wise from left to right on the pegboard, first using the dominant hand then the nondominant hand. Performance was rated as the time taken to complete the task with each hand.

### 2.5 Iowa Gambling task

IGT performance was tested in 21 CBTp+SC patients and 21 healthy participants. The goal was to maximize the monetary gain beyond the $2000 loan that participants were given to start with. Participants were informed that they could switch between decks at any time, since some decks were associated with more monetary loss than others and the aim was to avoid the “bad” decks. The task ended when 100 deck choices (trials) were made. Overall learning was measured by dividing the trials into five blocks of 20 and calculating the difference between blocks 1 and 5 in the number of advantageous (decks C and D) minus disadvantageous card selections (decks A and B).

The expectancy-valence model is an algorithm of three IGT performance-derived parameters (Busemeyer and Stout, 2002, Yechiam et al., 2005, Yechiam et al., 2007). It assumes that the following three parameters decide an individual׳s pattern of IGT performance (Yechiam et al., 2005):

(1) Attention to reward: this parameter assesses an individual׳s ability to guide his/her strategy based on gains or losses received during the task. High values on this parameter indicate greater attention to gains than to losses and a greater motivation towards reward. It is denoted by a utility function, *u*(*t*), that allows for different weights for gains and losses, where *t* is a given trial. It is calculated as,
  u(t)=Wwin(t)−(1−W)loss(t)
  where *win*(*t*) is the amount of money won on trial *t*; loss(*t*) is the amount of money lost on trial *t*; and *W* is a parameter that indicates the weight given to gains versus losses.
(2) Memory for past, relative to recent, outcomes: this parameter describes the degree to which expectancies of deck consequences reflect the influence of past experiences with particular decks. High values of this component indicate strong recency effects such that the most recent trials are more influential in determining the expectancy, whereas outcomes from past trials are discounted. On any trial *t*, the expected utility, *E*_*j*_, for deck *j* is equal to that endowed by the previous trials *E*_*j*_(*t−*1). Expected utility is then calculated as,
  $$E_{j}(t)=E_{j}(t-1)+\varphi [u(t)–E_{j}(t-1)]\delta_{j}(t)$$
  where *δ*_*j*_(*t*) reflects the change in expectancy if deck *j* was selected in trial *t* and *δ*_*j*_(*t*) is a weight associated with the chosen deck. When the expectancy is updated (*δ*_*j*_(*t*)=1), then a change occurs in the direction of the prediction error given by [*u*(*t*)−*E*_*j*_(*t*)].
(3) Choice consistency (low impulsivity): This parameter evaluates the reliability with which the decision-maker applies expectancies about each deck when making the card selection. High values of this parameter indicate that the deck with maximum expectancy will almost certainly be chosen on each trial, which indicates greater choice consistency and less impulsivity. It is assumed that the consistency, denoted by *θ* (*t*), changes as a function of experience:
  θ(t)=(t/10)c
  Reliability is represented by the choice consistency parameter, denoted as *c*. The parameter *c* controls the consistency of the choice probabilities and the expectancies.

### 2.6 MRI acquisition and measurements of regions of interest

Structural MRI brain scans (T1-weighted images in the axial plane with 1.5-mm contiguous sections, repetition time (TR)=18 ms, inversion time (TI)=450 ms, echo time (TE)=5.1 ms, flip angle=20° with one data average and a 256×256×128 voxel matrix) were acquired from a 1.5 T General Electric NV/i Signa System at the Maudsley Hospital, London.

A manual approach was chosen to perform region of interest (ROI) analysis based on the Cavalieri principle (Barta et al., 1997), because it provides an unbiased, direct and assumption-free estimate of the ROI (Sonmez et al., 2010). The Cavalieri principle (Barta et al., 1997) states that the volume of any object may be estimated by sectioning it with a set of parallel planes with uniform spacing and measuring the cross-sectional area of the object in each of the planes. The grid size is determined by trial and error. Volumes are obtained using different grid sizes, until the largest grid is determined that yields an estimated coefficient of error of less than 5% for volume estimates from any plane using the Gundersen formula (Gundersen and Jensen, 1987). Besides, a disadvantage with automated approaches, such as voxel-based morphometry, is that they are statistically derived and can therefore lack face validity (Kelemen et al., 1999).

Following the acquisition of the MR image, structural scans were aligned horizontally along the anterior commissure–posterior commissure plane and vertically along the interhemispheric fissure to correct for head tilt. ROI rating of the whole brain and OFC was performed using the MEASURE programme (Barta et al., 1997). To minimize bias during manual rating, all scans were coded to ensure that rating was carried out without knowledge of diagnostic status. Whole brain volume ratings were performed by a trained rater. The OFC was rated by A. Sapara and another trained rater. Interrater reliability was achieved if the trained rater agreed on the novice rater׳s ratings on 10 test brain scans. Left and right ROI measurements were derived from the total ROI measurements by performing plane cutaways left and right of the interhemispheric fissure.

#### 2.6.1 Whole brain volume

Rating of the whole brain was carried out mainly on the coronal view, while using the axial and sagittal views as reference. Existing criteria were followed to measure this region (DeLisi et al., 1995). The whole brain included the frontal, temporal, parietal and occipital lobes. It also included the subcortex, comprising the basal ganglia and thalamus. Tracings excluded the cerebellum, brain stem, ventricles, dura mater and cerebrospinal fluid surrounding the brain.

#### 2.6.2 Orbitofrontal cortex grey matter volume

**Fig. 1:**
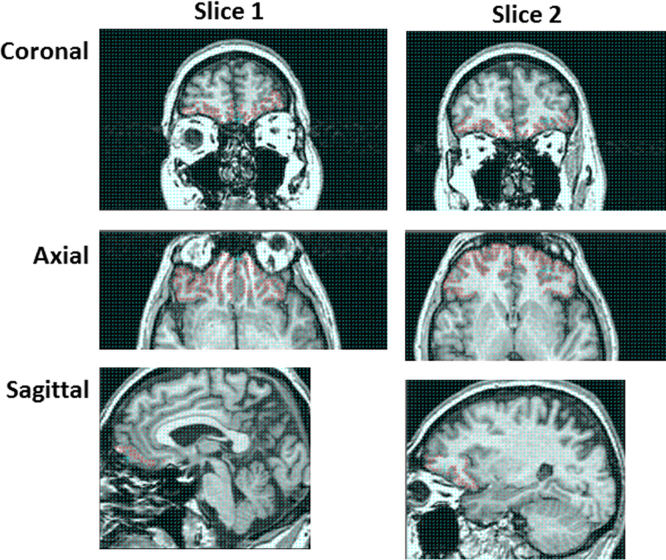
Rating of OFC grey matter as seen in the coronal, sagittal and axial views at two stereological points.

The OFC was measured using established criteria (Buchanan et al., 1998, Buchanan et al., 2004, Sapara et al., 2007; Kumari et al., 2009a; Ettinger et al., 2012). The OFC was defined superiorly by the horizontal ramus of the Sylvian fissure, medially by the superior rostral sulcus as it merges into the frontomarginal sulcus anteriorly, and posteriorly by the anterior olfactory sulcus (seen only in the midsagittal section) (Fig. 1).

### 2.7 Statistical analysis

#### 2.7.1 Data screening

Firstly, normality assumptions were tested on each variable of interest using the Kolmogorov–Smirnoff test. Variables with extreme scores (OFC total, left and right) were treated using a Windsor transformation of 5% and 95% at the lower and upper levels. Only one patient had outlier OFC GMV (because the boundaries the sulcal landmarks were not well defined).

#### 2.7.2 Comparison of demographic and OFC measures between patient and healthy participant groups

CBTp+SC and healthy participant groups were compared for age and years in education using one-way analysis of variance (ANOVA). Group difference in handedness was tested using a one-way ANOVA on the Edinburgh Handedness Inventory and a two-way ANOVA on the Grooved Pegboard test with handedness as the within-subjects variable and group as a between-subjects variable. The pre-to-post CBTp change in PANSS total and subscale scores was tested using ANOVA with time as the within-subjects variable. Total OFC GMV was compared between CBTp+SC and healthy participant groups using a one-way ANOVA. Left and right OFC GMVs were compared between CBTp+SC and healthy participant groups using ANOVA with hemisphere as the within-subjects variable and group as the between-subjects variable.

#### 2.7.3 Correlation between pre-therapy OFC measures and residual symptom change in patients

Residual symptom change in PANSS total, subscale (positive and negative symptoms and general psychopathology) and individual item scores was calculated using a linear regression. Here, residual symptom change was measured as the unstandardized predicted scores of observed follow-up symptom scores (criterion variable) and observed baseline symptom scores (predictor variable) (Siegle et al., 2006). This residual change score was then subtracted from one, so that higher scores meant more improvement. In both groups, OFC GMV was larger in the right than left hemisphere. Therefore OFC asymmetry was calculated as [(right OFC GMV−left OFC GMV)/(right OFC GMV+left OFC GMV)×100] (Robinson et al., 2004).

Partial correlations (one-tailed) were first performed between pre-therapy OFC variables (namely, total, left and right GMV) and residual symptom change in PANSS total and subscale scores while covarying for whole brain volume and age. Partial correlations were performed between rightward OFC asymmetry and symptom change scores with only age as a covariate. Whole brain volume was not used as a covariate in testing the symptom improvement correlates of the OFC rightward asymmetry index, because the calculation of the whole brain volume does not account for the posterior leftward asymmetry and the anterior rightward asymmetry in the Yarkelovian torque that occurs across the whole brain (Lemay and Kido, 1978, Pieniadz and Naeser, 1984). If a significant correlation at *p*≤0.05 emerged between an OFC measure and change in a particular PANSS subscale score, this was followed by partial correlations between the rank-ordered scores of the OFC measure and residual change scores in the individual items of that subscale while covarying for rank-ordered scores of whole brain volume and age. Rank-ordered scores of the correlates were used due to the ordinal nature of individual item data (1–7 range).

#### 2.7.4 Correlation between OFC measures and IGT performance

Partial correlations (one-tailed) were performed between OFC measures (total, left and right GMV) and IGT performance measures (overall learning, attention to reward, memory for past relative to recent outcomes and choice consistency/impulsivity) in the CBTp+SC (*n*=21 due to missing data) and healthy participant groups (*n*=21 due to missing data) while covarying for whole brain volume and age. Partial correlations were performed between rightward OFC asymmetry and IGT performance with only age as a covariate. Next, hierarchical regression analyses were performed to determine if IGT performance moderated the relation between symptom change and OFC GMV. Where both symptom change and IGT performance correlated significantly with an OFC GMV measure, a hierarchical regression analysis was performed with OFC GMV as the first step predictor, the product of OFC GMV and IGT performance as the second step predictor, and symptom change as the criterion variable.

The alpha level of statistical significance was set to *p*≤0.05. Statistical analyses were performed using SPSS, Version 21.

## 3 Results

### 3.1 Sample characteristics

Gender distribution was identical between groups (Table 1). The groups did not differ significantly in age and years in education. Groups were comparable on performance of both handedness measures. CBTp+SC patients showed an improvement of on average 13.8%, 14.3%, 10.4% and 12.5% on PANSS total score, positive symptom subscore, negative symptom subscore, and general psychopathology subscore, respectively. OFC GMV and IGT performance did not differ between patient and healthy groups. In both groups, OFC GMV was larger in the right than left hemisphere. There was no hemisphere-by-group interaction.

### 3.2 Association between pre-therapy OFC GMV, OFC rightward asymmetry and symptom improvement in patients

**Fig. 2:**
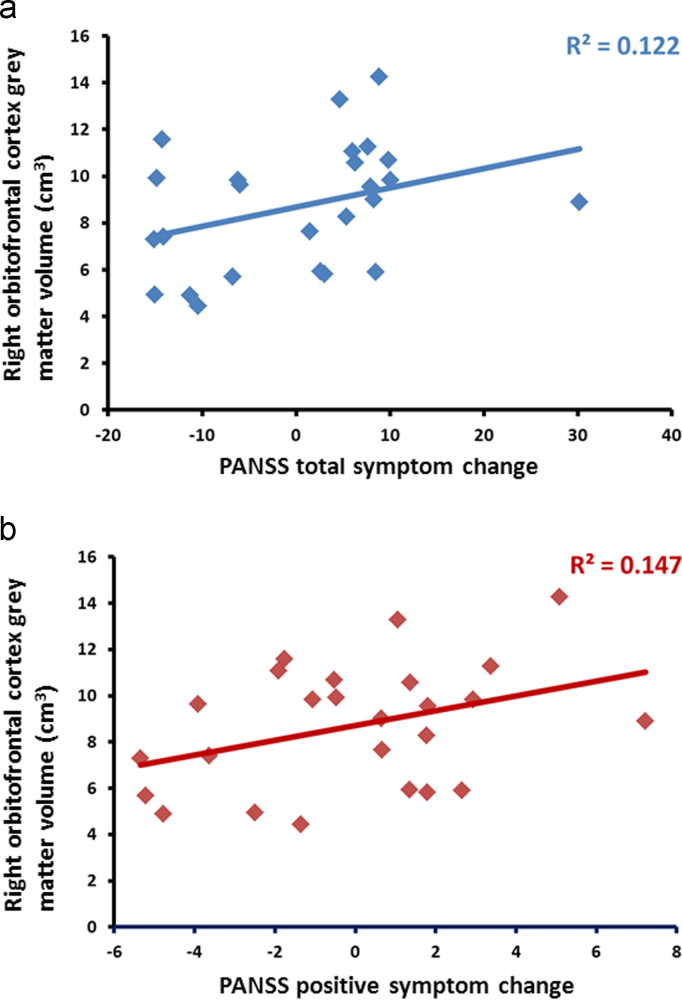
Scatter plot of right OFC grey matter volume in CBTp+SC patients with (a) PANSS total symptom change and (b) PANSS positive symptom change.

Greater pre-therapy total and right OFC GMV correlated with overall improvement in PANSS symptoms following CBTp (Table 2, Fig. 2). Greater pre-therapy total, left and right OFC GMV correlated with improvement in positive symptoms. Further exploration of the correlations between OFC GMV and individual positive symptom items revealed that improvement in hallucinations correlated positively with left OFC GMV (*r*=0.378, *p*=0.04). Improvement in persecutory beliefs correlated positively with total OFC GMV (*r*=0.414, *p*=0.02), right OFC GMV (*r*=0.378, *p*=0.04) and left OFC GMV (*r*=0.442, *p*=0.02). Greater right OFC GMV correlated with improvement in general psychopathology. Further exploration of the correlations between right OFC GMV and individual general psychopathology symptom items revealed that greater right OFC GMV correlated with improvement in insight (*r*=0.416, *p*=0.02) and disturbed volition (*r*=0.385, *p*=0.03).

Greater rightward OFC asymmetry in the CBTp+SC group correlated with improvement in total symptoms, negative symptoms and general psychopathology. Further examination of the OFC asymmetry-negative symptom correlation through individual negative symptom item correlations revealed that rightward OFC asymmetry correlated with improvement in blunted affect (*ρ*=0.405, *p*=0.02) and stereotyped thinking (*ρ*=0.374, *p*=0.04). Correlations between rightward OFC asymmetry and individual general psychopathology items showed that rightward OFC asymmetry correlated with improvement in tension (*ρ*=0.441, *p*=0.02), disorientation (*ρ*=0.423, *p*=0.02), impulse control (*ρ*=0.416, *p*=0.02), and active social avoidance (*ρ*=0.506, *p*=0.01).

### 3.3 Association between pre-therapy OFC GMV and IGT performance in patients

Greater total and left OFC GMV correlated with lower pre-therapy IGT attention to reward (Table 2). In healthy participants, greater OFC rightward asymmetry related to better overall IGT learning, while greater total, left and right OFC GMV related to lower consistency in making choices from a certain deck. Given that total GMV and left OFC GMV correlated with both positive symptom change and IGT attention to reward, hierarchical regression analyses were performed to test the moderation of the relation between total or left OFC GMV and positive symptom change by IGT attention to reward. Before the hierarchical regression analysis was performed, each variable was mean-centred (Jaccard et al., 1990). Positive symptom change was entered as the criterion variable, total or left OFC GMV as the predictor in the first step and the product of OFC GMV and IGT attention to reward as the predictor in the second step.

In the regression analysis predicting total OFC GMV, IGT attention to reward and positive symptoms were entered as predictors. The first model was statistically significant, *F* (1,19)=4.328, *p*=0.05; and it explained 18.6% of the variance (*R*=0.431, adjusted *R*^2^=0.186). The standardized beta coefficient (*β*) for total GMV was statistically significant (*β*=0.431, *p*=0.05). The second model approached statistical significance, *F* (2,18)=3.385, *p*=0.06; total OFC GMV, and the interaction between total OFC GMV and IGT attention to reward explained 27.3% of the model variance (*R*=0.523, adjusted *R*^2^=0.193, *R*^2^ change=0.088, *F* change=2.175, *p*=0.158), indicating that IGT attention to reward had an additive effect (8.7%) on explaining variance in CBTp responsiveness. *β* for total OFC GMV was significant (*β*=0.449, *p*=0.04, partial *r*=0.465), but β for the interaction term was not significant (*β*=0.297, *p*=0.16, partial *r*=0.328). The percentage of variance in positive symptom change explained by total OFC GMV and the interaction term were 21.6% and 10.7%, respectively.

In the regression analysis predicting total OFC GMV, IGT attention to reward and positive symptoms were entered as predictors. The first model approached statistical significance, *F* (1,19)=3.997, *p*=0.06, and explained 17.4% of the variance (*R*=0.413, adjusted *R*^2^=0.130). The standardized beta coefficient (*β*) for left OFC GMV approached statistical significance (*β*=0.417, *p*=0.06). The second model was significant, *F* (2,18)=3.505, *p*=0.05; left OFC GMV and the interaction between left OFC GMV and IGT attention to reward explained 28% of the model variance (*R*=0.529, adjusted *R*^2^=0.20, *R*^2^ change=0.107, *F* change=2.664, *p*=0.120), again indicating that IGT attention to reward had an additive effect (10.6%) on explaining variance in CBTp responsiveness. *β* for left OFC GMV was significant (*β*=0.462, *p*=0.03, partial *r*=0.475), but *β* for the interaction term was not significant (*β*=0.329, *p*=0.12, partial *r*=0.359). The percentages of variance in positive symptom change explained by left OFC GMV and the interaction term were 22.6% and 12.9%, respectively.

## 4 Discussion

The aim of the study was to determine whether greater pre-therapy OFC GMV would be associated with better CBTp responsiveness in patients with schizophrenia because of the OFC׳s role in emotional decision-making and cognitive flexibility. As hypothesized, greater pre-therapy OFC GMV (total, left and right) was associated with greater post-therapy improvement in positive symptoms. In particular, greater pre-therapy left OFC GMV was associated with improvement in hallucinations, and greater pre-therapy total, right and left OFC GMV were associated with improvement in persecutory beliefs. Furthermore, greater pre-therapy total and left OFC GMV were associated with lower IGT attention towards reward. However, the relation between total or left OFC GMV and positive symptom improvement following CBTp was not moderated by IGT attention to reward. Instead, IGT attention to reward had an additive effect on positive symptom improvement, because IGT attention to reward increased the prediction of positive symptom improvement by total and left OFC GMV. In healthy participants, greater OFC GMV was associated with lower IGT choice consistency, suggesting that greater OFC GMV facilitates a less perseverative response style and more flexibility contingent on reward experiences associated with a deck. This finding is consistent with evidence that the OFC is involved in cognitive flexibility-related functions (Bechara et al., 1994, Bechara et al., 2000, Schoenbaum et al., 2009).

A positive association between rightward OFC asymmetry and CBTp responsiveness was found. Greater rightward OFC GMV asymmetry correlated with improvement in overall symptoms and negative symptoms, and in turn with improvement in blunted affect and stereotypical thinking. Greater rightward OFC GMV asymmetry also correlated with improvement in general psychopathology, specifically with improvement in tension, disorientation, impulse control and active social avoidance.

### 4.1 Association between OFC GMV and improvement in positive symptoms following CBTp

The OFC׳s association with positive symptom change and better IGT performance is consistent with evidence that greater OFC GMV is associated with better symptom insight and foresight in patients with schizophrenia (Eack et al., 2008, Sapara et al., 2007, Shad et al., 2006), because better insight predicts positive symptom improvement at 12-month follow-up (Gumley et al., 2014). Schizophrenia patients with pronounced positive symptoms resemble patients with OFC lesion in neither showing regret nor weighing the negative consequences of their choices during emotional decision-making (Larquet et al., 2010). Good cognitive insight – the patients׳ ability to reflect on their experiences and recognize that their conclusions may be incorrect (Beck et al., 2004) – predicts better CBTp outcome (Perivoliotis et al., 2010, Premkumar et al., 2011). The present study׳s findings are consistent with the OFC׳s roles in emotional decision-making and cognitive flexibility in terms of being able to regulate one׳s emotions when weighing up the consequences of one׳s behaviour and this role may be relevant to greater CBTp efficacy. However, the present study׳s findings do not directly confirm an effect of emotional decision-making on CBTp responsiveness, because IGT attention to reward on its own did not relate to symptom improvement, nor did it moderate the association between left OFC GMV and CBTp-related symptom improvement, but it increased the strength of the association between left OFC GMV and symptom improvement.

In the present study, greater OFC GMV related to improvement in two positive symptoms, namely hallucinations and persecutory beliefs. Enhanced OFC function in terms of emotional decision-making and being able to respond more flexibly to advice given during therapy may account for the observed improvement in these symptoms. Poor belief flexibility relates to persecutory delusions under conditions of negative affect (Freeman et al., 2008, Garety et al., 2013), which CBTp aims to modify (Freeman et al., 2012). Patients with active delusions exhibit jumping-to-conclusions reasoning, whereby they use less evidence to confirm their judgements compared to healthy participants (Warman and Martin, 2006). Patients with either current or remitted delusions are less likely to express belief flexibility and more likely to be preoccupied with generally held beliefs, but not delusional or idiosyncratic beliefs, than healthy participants (Colbert et al., 2010). In a previous report of the clinical predictors of CBTp responsiveness in the present study׳s cohort (Premkumar et al., 2011), we found that greater self-reflectiveness about one׳s anomalous experiences before therapy predicted greater CBTp responsiveness. The relation between greater left OFC GMV and improvement in persecutory beliefs following CBTp observed in the present study therefore provides a neurobiological explanation for why cognitive flexibility would predict CBTp responsiveness.

### 4.2 Association between rightward OFC asymmetry and improvement in negative symptoms and general psychopathology follow CBTp

The patients showed preserved rightward OFC asymmetry as well as handedness. Hemispheric difference in OFC gyrification is normal relative to other prefrontal regions in schizophrenia patients (Palaniyappan et al., 2011). The association between OFC asymmetry and age of onset was negligible even though a lack of total frontal lobe GMV asymmetry correlated with an earlier age of onset in schizophrenia patients (Maher et al., 1998). During brain development, the brain twists around its longitudinal axis so that the left occipital lobe and right frontal lobe develop greater protrusions than the corresponding lobe in the opposite hemisphere (Lemay and Kido, 1978, Pieniadz and Naeser, 1984). This twisting of the brain is referred to as the Yakovlevian torque. Asymmetry development continues until the end of adolescence (Feinberg, 1982, Crow, 1990, Buchsbaum et al., 1992). A loss of cerebral torque is associated with the onset of schizophrenia (Maher et al., 1998), whereas greater cerebral torque is associated with fewer negative symptoms (Bilder et al., 1994), better long-term social functioning and remission from symptoms at the early stage of schizophrenia (Robinson et al., 2004). Normal asymmetry may reflect intact functional connectivity. Reduced asymmetry in the Broca׳s area during verbal fluency was accompanied by reduced interhemispheric functional connectivity in schizophrenia patients (Bleich-Cohen et al., 2009, Bleich-Cohen et al., 2012). In the present study, the association between greater pre-therapy rightward OFC asymmetry and improvement in negative symptoms and general psychopathology following CBTp could suggest that the normal development of OFC asymmetry, and in turn better functional connectivity, may preserve OFC functions that are relevant to CBTp responsiveness. Normal asymmetry development in the OFC may be important for the long-term efficacy of therapies that help to alleviate distress with psychotic symptoms and improve social functioning.

### 4.3 Limitations

Studies previously examining the clinical correlates of OFC subregions have found greater left medial OFC volume to correlate with lower impulsivity (Schiffer et al., 2010) and poorer clinical insight (Shad et al., 2006) in schizophrenia patients. In the present study, only total left and right volumes of the OFC were measured and not their subregions. Therefore, it could not be ascertained whether GMV of specific OFC subregions accounted for the OFC׳s relation to positive symptom improvement. Another limitation was the small sample size of the participant groups, so that the CBTp group may not have had sufficient power to generate correlations with large effect sizes. For this reason, an adjustment for Type I errors was not made. Thirdly, an active control intervention (e.g., supportive counselling) was not used as a comparator in this study; thus, a similar association between pre-CBTp OFC parameters and responsiveness to other psychological therapies cannot be ruled out.

### 4.4 Summary and conclusion

Greater OFC GMV and rightward asymmetry were associated with greater symptom improvement following CBTp, indicating that the integrity of the OFC in supporting good emotional decision-making is conducive for responsiveness to CBTp. Given that changes in both of these OFC measures closely relate to the onset of psychosis (Pantelis et al., 2003, Borgwardt et al., 2008, Sheng et al., 2013), normal development of OFC may be a defining factor in determining CBTp responsiveness by preserving its functions, namely emotional decision-making and belief flexibility.

## Role of funding source

Funding for this study was provided by the Wellcome Trust, UK [067427/z/02/z, Wellcome Senior Fellowship to VK]. V. Kumari and A. Simmons are in part supported by the NIHR Biomedical Research Centre for Mental Health at South London and Maudsley NHS Foundation Trust and King׳s College London, Institute of Psychiatry, Psychology and Neuroscience. The Wellcome Trust had no further role in study design; in the collection, analysis and interpretation of data; in the writing of the report; and in the decision to submit the manuscript for publication.

## Figures and Tables

**Table 1:** Demographic, pre-therapy orbitofrontal cortex grey matter volume, pre- and post-therapy PANSS symptoms, and pre-therapy neuropsychological performance (mean, S.D. and range) in CBTp+SC patients (*n*=25) and healthy participants (*n*=25).

Characteristic	CBTp+SC patients		Healthy participants		Model	*F* (d.f.)	*P* value
Sex: male/female (*n*)	17/8		17/8		Group		
Age in years	35.56, 8.11, 21–49		33.72, 12.64, 20–65		Group	0.62 (1,48)	0.43
Years in education	13.88, 3.07, 8–20		15.08, 2.46, 10–20		Group	1.86 (1,48)	0.18
Edinburgh Handedness Inventory	9.42, 1.09, 6–10.0		8.64, 1.40, 5.5–10.0		Group	3.22 (1,48)	0.18
Grooved pegboard test	Dominant	Non-dominant	Dominant	Non-dominant			
	76.04, 19.57, 5.00–110.62	87.14, 21.66, 6.20–111.27	63.41, 13.96, 49.24–120.09	76.22, 20.80, 46.63–117.85	Hand×Group	0.13 (1,48)	0.71
Total OFC GMV (cm^3^)	17.17, 4.43, 10.44–24.40	19.66, 6.73, 9.25–33.85			Group	3.77 (2)	0.15
Left OFC GMV (cm^3^)	8.54, 2.10, 4.74–12.19	9.43, 3.50, 3.96–16.81					
Right OFC GMV (cm^3^)	8.70, 2.66, 4.44–14.26	10.23, 3.34, 4.82–17.57			Hemisphere×Group	1.92 (2,66)	0.15

Symptoms (PANSS)	Baseline	Follow-up					
Positive	18.08, 4.78, 7–25	14.84, 4.17, 7–23^a^	-		Time	14.11 (1,24)	0.001
Negative	17.88, 4.37, 8–27	15.48, 4.20, 8–24^a^	-		Time	9.61 (1,24)	0.005
General psychopathology	33.16, 7.18, 22–56	28.32, 7.14, 16–41^a^	-		Time	9.93 (1,24)	0.004
Total	69.12, 13.61, 43–108	58.64, 14.49, 31–78^a^	-		Time	15.2 (1,24)	0.001
IGT overall learning^b^	7.81, 16.61, −16.0 to 36.0		10.00, 13.04, −16 to 29.0		Group	3.30 (2)^c^	0.19
IGT attention to reward^b^	0.46, 0.39, 0–1.0		0.41, 0.33, 0–1.0		Group	0.09 (2)^c^	0.96
IGT memory for past relative to recent outcomes^b^	0.37, 0.39, 0–1.0		0.22, 0.69, 0–1.0		Group	1.14 (2,57)	0.33
IGT choice consistency^b^	0.14, 2.58, −5.0 to 5.0		0.41, 2.06, −5 to 3.39		Group	0.45 (2,57)	0.64

GMV: grey matter volume, IGT: Iowa Gambling Task, OFC: orbitofrontal cortex.

a Significant reduction in symptom severity at follow-up.

b Group comparisons based on pre-therapy IGT performance in 21 CBTp+SC patients and 21 healthy participants.

c Kruskal–Wallis test performed due to heterogeneity of variance between groups.

**Table 2:** Partial correlations (p) between OFC grey matter volume or OFC asymmetry and residual symptom change and IGT performance after covarying for whole brain volume and/or age.

	CBTp+SC group				Healthy participant group			
Baseline ROI variable	Total	Left	Right	Asymmetry	Total	Left	Right	Asymmetry
PANSS Total	**0.439 (0.03)**	0.302 **(**0.10)	**0.518 (0.01)**	**0.537 (0.01)**	–	–	–	–
PANSS Positive	**0.530 (0.01)**	**0.496 (0.01)**	**0.553 (0.01)**	0.308 **(**0.09)	–	–	–	–
PANSS Negative	0.306 **(**0.10)	0.146 **(**0.28)	0.379 **(**0.05)	**0.542 (0.01)**	–	–	–	–
PANSS General psychopathology	0.347 **(**0.07)	0.209 **(**0.20)	**0.440 (0.03)**	**0.482 (0.02)**	–	–	–	–
Pre-therapy IGT overall learning^a^	0.177 **(**0.23)	0.126 **(**0.30)	0.253 **(**0.15)	0.280 **(**0.12)	0.074 (0.38)	−0.008 (0.49)	0.161 (0.26)	**0.417 (0.04)**
Pre-therapy IGT attention to reward^a^	−**0.439 (0.03)**	−**0.472 (0.02)**	−0.351 **(**0.07)	−0.061 **(**0.40)	−0.050 (0.42)	−0.085 (0.37)	−0.010 (0.48)	0.160 (0.26)
Pre-therapy IGT memory for past relative to recent outcomes^a^	−0.087 **(**0.36)	−0.121 **(**0.31)	−0.075 **(**0.38)	0.051 **(**0.41)	0.215 (0.20)^b^	0.216 (0.19)^b^	0.286 (0.12)^b^	−0.034 (0.44)^b^
Pre-therapy IGT choice consistency^a^	−0.100 **(**0.34)	−0.090 **(**0.36)	−0.077 **(**0.38)	−0.023 **(**0.46)	−**0.501 (0.02)**	−**0.466 (0.03)**	−**0.519 (0.01)**	0.035 (0.44)

Partial correlations were performed between OFC GMV and PANSS total and subscale scores and IGT performance while covarying for whole brain volume and age; partial correlations were performed between OFC rightward asymmetry and PANSS total and subscale scores and IGT performance while covarying for age.

a Correlations based on 21 CBTp+SC patients and 21 healthy participants.

b Spearman partial correlation was performed for IGT memory for past relative to recent outcomes because data transformation could not normalize data.
